# Clinical outcomes of locally advanced esophageal neuroendocrine carcinoma treated with chemoradiotherapy

**DOI:** 10.1002/cam4.2708

**Published:** 2019-12-03

**Authors:** Yoshitaka Honma, Kengo Nagashima, Hidekazu Hirano, Hirokazu Shoji, Satoru Iwasa, Atsuo Takashima, Natsuko Okita, Ken Kato, Narikazu Boku, Naoya Murakami, Kouji Inaba, Yoshinori Ito, Jun Itami, Jun Kanamori, Junya Oguma, Hiroyuki Daiko

**Affiliations:** ^1^ Gastrointestinal Medical Oncology Division National Cancer Center Hospital Tokyo Japan; ^2^ The Institute of Statistical Mathematics Research Center for Medical and Health Data Science Tokyo Japan; ^3^ Department of Radiation Oncology National Cancer Center Hospital Tokyo Japan; ^4^ Department of Radiation Oncology Showa University School of Medicine Tokyo Japan; ^5^ Esophageal Surgery Division National Cancer Center Hospital Tokyo Japan

**Keywords:** chemoradiotherapy, esophageal neuroendocrine carcinoma, etoposide, multidisciplinary treatment, platinum

## Abstract

**Background:**

Neuroendocrine carcinoma (NEC) arising from the esophagus (EsoNEC) is extreme rare, accounting for approximately 1% of esophageal cancer. Even for localized NEC, multidisciplinary approach including chemotherapy is recommended in treatment guidelines because of its high rates of systemic recurrence. However, it is controversial whether adding surgery or radiotherapy is appropriate local treatment for EsoNEC. There have been few reports regarding the clinical outcomes of definitive chemoradiotherapy (dCRT) for EsoNEC. The purpose of this study was to clarify the survival outcome of patients with locally advanced EsoNEC treated with dCRT.

**Methods:**

Clinical outcomes, feasibility, and prognostic factors of patients with locally advanced EsoNEC treated with radiotherapy (60 Gy/30 fraction) in combination with platinum plus etoposide (CE‐RT) or cisplatin plus 5‐fluorouracil (CF‐RT) at the National Cancer Center Hospital from 2001 to 2017 were retrospectively analyzed.

**Results:**

A total of 22 patients were identified as the subjects of this study. The overall response rate and clinical complete remission rate in all patients were 86.4% and 77.3%, respectively. The median progression‐free survival and median survival time in all patients were 12.7 and 37.5 months, associated with a 5‐year survival rate of 45.4%. Patients treated with CE‐RT experienced more hematological adverse events, especially in neutropenia (≥grade 3) and febrile neutropenia(≥grade 3), but achieved more long‐term progression‐free survival than with CF‐RT.

**Conclusions:**

Definitive chemoradiotherapy can be considered as an important treatment option for locally advanced esophageal neuroendocrine carcinoma.

## Graphical abstract

Definitive chemoradiotherapy can be considered as an important treatment option as well as surgery for locally advanced esophageal neuroendocrine carcinoma.

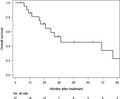

## 1 INTRODUCTION

Neuroendocrine carcinoma (NEC) is a high‐grade neoplasm with pathological neuroendocrine features, such as expression of chromogranin A and synaptophysin. In the current World Health Organization (WHO) classification of tumors of the digestive system, NEC is defined as poorly differentiated carcinoma with a mitotic count of >20 per 10 high power field and/or a proliferation index >20%, mainly consisting of small cell carcinoma and large cell carcinomas.^1^

NEC arising from the esophagus (EsoNEC) is extremely rare, accounting for approximately 1% of all esophageal malignant diseases, and its incidence is reportedly more common in Asian countries than in Western countries.^3^, ^4^, ^5^

In “typical” esophageal cancer such as adeno‐ or squamous cell carcinoma, perioperative chemotherapy or chemoradiotherapy before or after surgery is the standard treatment, and definitive chemoradiotherapy (dCRT) is performed alternatively as an optional treatment.^6^ Even for localized NEC, chemotherapy plays an important role in the multidisciplinary approach recommended in treatment guidelines because of its high rates of systemic recurrence and poor prognosis.^1^, ^2^

Considering its high sensitivity to radiotherapy, which is demonstrated by small cell lung cancer, and the invasiveness of radical esophagectomy, which may compromise the patient's condition, it is controversial whether surgery or radiotherapy are appropriate local therapies for EsoNEC.^7^, ^8^, ^9^, ^10^ However, the clinical outcomes of dCRT for EsoNEC are not well known because of its rarity. The purpose of this study was to clarify the clinical outcomes of locally advanced EsoNEC treated with dCRT.

## 2 MATERIALS AND METHODS

### 2.1 Patient selection

We retrospectively reviewed the medical records of patients with locally advanced EsoNEC who were initially treated with dCRT at the National Cancer Center Hospital (NCCH) from 2001 to 2017. The subjects of this study were extracted from our consecutive patients’ database. Clinical staging was determined based on a multidisciplinary team conference based on the findings of upper gastrointestinal endoscopy (UGI), endoscopic ultrasonography, and cervico‐thoracic‐abdominal thin slice computed tomography (CT). Neck ultrasonography and ^18^F‐fluorodeoxyglucose positron emission tomography were performed if necessary. Patients with histology other than neuroendocrine carcinoma, Stage IA disease, distant metastases other than supraclavicular lymph node (SCLN), simultaneous advanced malignancy, or without detailed follow‐up data were excluded. This retrospective study was approved by the institutional review boards of NCCH (2012‐268).

### 2.2 Treatment

Subjects were treated with two courses of chemotherapy with (a) cisplatin (80 mg/m^2^, day 1) plus etoposide (100 mg/m^2^, days 1－3) administered every 3－4 weeks; (b) carboplatin (area under the curve: 5, day 1) plus etoposide (80 mg/m^2^, days 1－3) administered every 3－4 weeks; or cisplatin (70 mg/m^2^, day1) plus 5‐fluorouracil (700 mg/m^2^, days 1－4) administered every 4 weeks. Patients were divided into two groups according to the treatment regimen; CE‐RT (cisplatin or carboplatin plus etoposide) and CF‐RT (cisplatin plus 5‐fluorouracil) groups.

Radiotherapy was delivered concurrently with chemotherapy using megavoltage (≥6 MV) X‐rays; a total dose of 60 Gy was administered in 30 fractions without break. The clinical target volume (CTV) for 60‐Gy irradiation included the primary tumor plus a 2‐3 cm cranio‐caudal margin, and the metastatic lymph nodes plus a 0‐1‐cm margin. Planning target volume was defined as CTV plus 5‐ to 20‐mm margins. Basically, elective nodal irradiation (40 Gy) was performed even in T4 case. The regions for elective nodal irradiation were planned by the judgment of radiation oncologist and mainly included the mediastinal and perigastric/celiac lymph nodes, and in some cases bilateral cervical lymph nodes. Basically, the radiation field was determined by three‐dimensional planning using CT.

### 2.3 Data collection and statistical analyses

Performance status (PS) was evaluated according to the Eastern Cooperative Oncology Group criteria. Clinical staging was classified according to the 7th edition of the UICC‐TNM classification because its 8th edition focuses on only squamous cell carcinoma and adenocarcinoma. Adverse events during CRT were evaluated according to Common Terminology Criteria for Adverse Events (CTCAE) version 4.0. A clinical complete remission (cCR) after dCRT was defined when (a) no residual tumor was detected by UGI and CT and (b) no malignant cell was detected in endoscopic biopsy specimens. Overall survival (OS) was defined as the time from the first date of the treatment to death (any cause) or censored at the last date of confirmed survival. Progression‐free survival (PFS) was defined as the time from the first date of the treatment to the date of the first documentation of disease progression or death (any cause), whichever came first, or censored at the last date of confirming survival without disease progression. For patients who underwent salvage esophagectomy for remnant disease without disease progression after dCRT, incomplete (R1/2) surgery or disease recurrence after curative (R0) surgery were counted as PFS events. Disease progression was divided into locoregional progression (LP) inside of the radiation field and distant progression (DP) outside the radiation field.

The median follow‐up time was estimated using the reverse Kaplan‐Meier method. Survival curves were drawn using the Kaplan‐Meier method and compared by the log‐rank test. Univariate analyses for detecting prognostic factors were performed with Cox regression models. All statistical analyses were performed using the SAS software (version 9.4 SAS Institute, Inc).

## 3 RESULTS

### 3.1 Patients' characteristics

Of 5483 patients with primary esophageal cancer treated at the NCCH from January 2001 to December 2017, 45 with EsoNEC were identified. After excluding 23 patients who had distant metastases other than supraclavicular lymph node (N = 20) or Stage IA (N = 1) and concurrent active malignancies (N = 2), a total of 22 patients were included in the study. Patient characteristics are shown in Table 1.

**Table 1:** Patient characteristics

Characteristic	All (n = 22)	CE‐RT (n = 17)	CF‐RT (n = 5)	*P*‐value
Median age (range)	62.0 (51‐81)	66.0 (51‐81)	62.0 (59‐74)	.644
Age ‐ No. (%)				.323
<65	12 (54.5)	8 (47.1)	4 (80.0)	
≥65	10 (45.5)	9 (52.9)	1 (20.0)	
Gender ‐ No. (%)				.266
Male	16 (72.7)	11 (64.7)	5 (100.0)	
Female	6 (27.3)	6 (35.3)	0 (0.0)	
Performance status (PS) ‐ No. (%)				1.000
0	11 (50.0)	8 (47.1)	3 (60.0)	
1	11 (50.0)	9 (52.9)	2 (40.0)	
Tumor location ‐ No. (%)				.289
Upper (Ut)	3 (13.6)	3 (17.6)	0 (0.0)	
Middle (Mt)	9 (40.9)	8 (47.1)	1 (20.0)	
Lower (Lt)	10 (45.5)	6 (35.3)	4 (80.0)	
Clinical T stage (cT) ‐ No. (%)				.630
T1	1 (4.5)	1 (5.9)	0 (0.0)	
T2	4 (18.2)	4 (23.5)	0 (0.0)	
T3	11 (50.0)	7 (41.2)	4 (80.0)	
T4	6 (27.3)	5 (29.4)	1 (20.0)	
Clinical N stage (cN) ‐ No. (%)				.361
N0	2 (9.1)	2 (11.8)	0 (0.0)	
N1	13 (59.1)	9 (52.9)	4 (80.0)	
N2	5 (22.7)	5 (29.4)	0 (0.0)	
N3	2 (9.1)	1 (5.9)	1 (20.0)	
Clinical M stage (cM) ‐ No. (%)				.411
M0	20 (90.9)	16 (94.1)	4 (80.0)	
M1	2 (9.1)	1 (5.9)	1 (20.0)	
Clinical Stage ‐ No. (%)				.730
Stage IB	2 (9.1)	2 (11.8)	0 (0.0)	
Stage IIA/B	3 (13.6)	3 (17.6)	0 (0.0)	
Stage IIIA/B/C	15 (68.2)	11 (64.7)	4 (80.0)	
Stage IV	2 (9.1)	1 (5.9)	1 (20.0)	
Histology ‐ No. (%)				.056
Small cell NEC	12 (54.5)	9 (52.9)	3 (60.0)	
Large cell NEC	3 (13.6)	1 (5.9)	2 (40.0)	
Unclassified NEC	7 (31.8)	7 (41.2)	0 (0.0)	
Chromogranin A staining ‐ No. (%)				.226
Positive	13 (59.1)	11 (64.7)	2 (40.0)	
Negative	8 (36.4)	6 (35.3)	2 (40.0)	
Unknown	1 (4.5)	0 (0.0)	1 (20.0)	
Synaptophysin staining ‐ No. (%)				.117
Positive	19 (86.4)	16 (94.1)	3 (60.0)	
Negative	2 (9.1)	1 (5.9)	1 (20.0)	
Unknown	1 (4.5)	0 (0.0)	1 (20.0)	
CD56 staining ‐ No. (%)				1.000
Positive	16 (72.7)	12 (70.6)	4 (80.0)	
Negative	3 (13.6)	3 (17.6)	0 (0.0)	
Unknown	3 (13.6)	2 (11.8)	1 (20.0)	
Albumin ‐ No. (%)				1.000
<4.0 g/dL	6 (27.3)	5 (29.4)	1 (20.0)	
≥4.0 g/dL	16 (72.7)	12 (70.6)	4 (80.0)	
White blood cell counts ‐ No. (%)				1.000
≤8000/mm^3^	15 (68.2)	11 (64.7)	4 (80.0)	
>8000/mm^3^	7 (31.8)	6 (35.3)	1 (20.0)	
NSE level ‐ No. (%)				.603
≤15 ng/mL	12 (60.0)	8 (53.3)	4 (80.0)	
>15 ng/ mL	8 (40.0)	7 (46.7)	1 (20.0)	
CRT regimen ‐ No. (%)				—
Cisplatin + Etoposide ‐ RT	16 (72.7)	16 (94.1)	0 (0.0)	
Carboplatin + Etoposide ‐ RT	1 (4.5)	1 (5.9)	0 (0.0)	
Cisplatin + 5‐fluorouracil ‐ RT	5 (22.7)	0 (0.0)	5 (100.0)	

A total of 17 out of 22 (77.3%) patients received radiotherapy concurrent with platinum and etoposide (CE‐RT group). The other 5 patients received cisplatin plus 5‐fluorouracil (CF‐RT group). Five of the 17 patients in the CE‐RT group had Stage IB or II disease, while all patients in the CF‐RT group had more advanced (≥Stage III) disease. Only 1 patient treated with CF‐RT received induction chemotherapy with cisplatin plus etoposide before dCRT. Radiation therapy for 1 patient was planned by two‐dimensional CT.

### 3.2 Tumor response

A total of 20 out of 22 (90.9%) patients had target lesions, 17 (85.0%) obtained tumor response while cCR was achieved in 15 (75.0%) patients. On the other hand, the remaining 2 patients without target lesion achieved cCR at the primary tumors, therefore the overall cCR rate in was 77.3% (17/22). cCR rate by treatment regimen were 70.5% in the CE‐RT group (12/17), and 100% in the CF‐RT group (5/5), respectively.

### 3.3 Survival outcome and prognostic factor analyses

**Figure 1:**
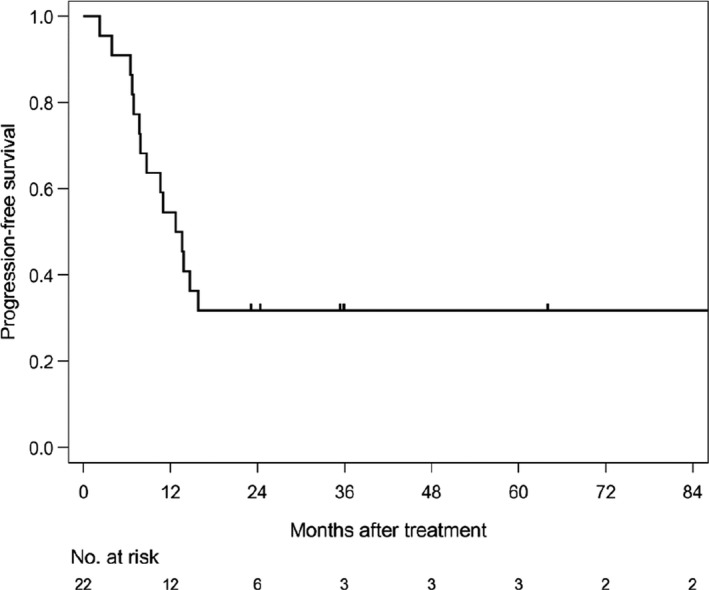
Progression‐free survival in all patients

**Figure 2:**
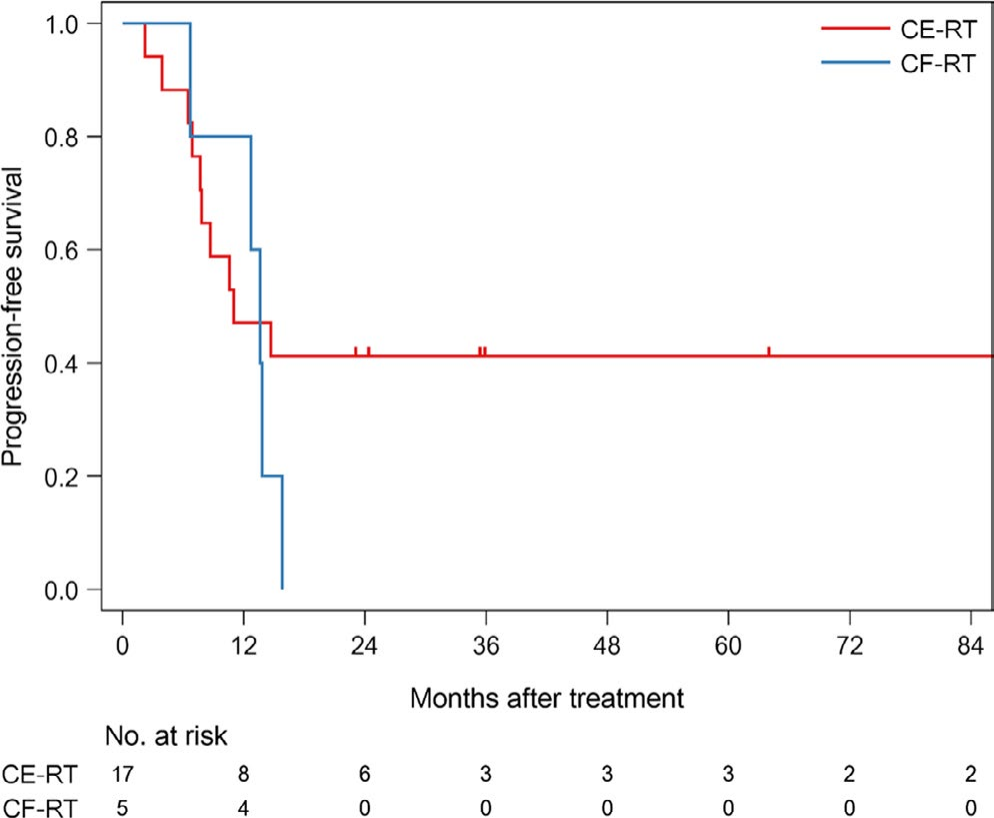
Progression‐free survival by CRT regimen

**Figure 3:**
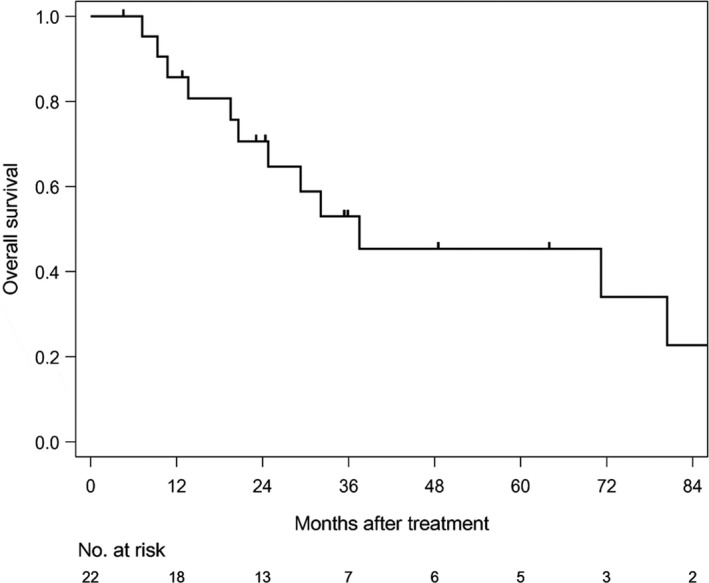
Overall survival in all patients

**Figure 4:**
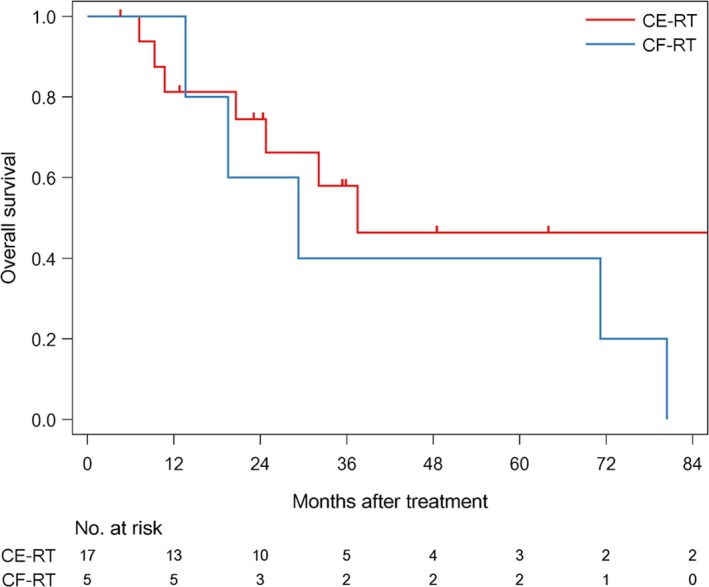
Overall survival by CRT regimen

The median follow‐up time of survivors was 35.6 months (range: 4.6‐186.6). The median PFS was 12.7 months (1/3/5 year‐PFS: 54.5/31.8/31.8%) in all patients (Figure 1). The median PFS was 11 months (1/3/5 year‐PFS; 47.1/41.2/41.2%) in patients treated with CE‐RT, and 13.6 months (1/3/5 year‐PFS: 80/0/0%) in patients treated with CF‐RT (Figure 2). The median survival time (MST) was 37.5 months (1/3/5 year‐OS: 85.7/52.9/45.4%) in all patients (Figure 3). MST was 37.5 months (1/3/5 year‐OS; 81.3/57.9/46.3%) in patient treated with CE‐RT, and 29.3 months (1/3/5 year‐OS: 100/40/40%) in patients treated with CF‐RT (Figure 4). Although no significant survival difference was observed between the two chemotherapy regimens (Figure 2: *P* = .389, Figure 4: *P* = .277), patients treated with CE‐RT achieved more long‐term progression‐free survival than with CF‐RT (8 in CE‐RT, 0 in CF‐RT).

Univariate analysis indicated that patients with depth of primary lesion (T3‐4) and high WBC counts (>8000/mm^3^) tended to have poor PFS (Table 2), whereas SCLN metastases (M1) was associated with poor OS (Table 3). No long‐term survival was achieved in patients with SCLN metastases.

**Table 2:** Univariate analysis for PFS with Cox regression models and summary statistics

Variable	Univariate analysis (N = 22)					
	HR	95% CI		*P*‐value	1/3/5 y ‐ PFS [%]	Median [mo]
CRT regimen						
CE‐RT	Reference				47.1/41.2/41.2	11.0
CF‐RT	1.680	0.571	4.942	0.346	80.0/0.0/0.0	13.6
Age						
<65	Reference				66.7/33.3/33.3	14.2
≥65	1.297	0.467	3.602	0.618	40.0/30.0/30.0	9.7
Gender						
Male	Reference				50.0/18.8/18.8	11.9
Female	0.371	0.091	1.512	0.166	66.7/66.7/66.7	NR
PS						
0	Reference				63.6/36.4/36.4	13.8
1	1.481	0.536	4.093	0.449	45.5/27.3/27.3	11.0
cT						
T1‐2	Reference				80.0/80.0/80.0	NR
T3‐4	4.760	0.827	27.414	0.081	47.1/17.6/17.6	11.0
cN						
N0‐1	Reference				60.0/33.3/33.3	13.8
N2‐3	1.264	0.432	3.702	0.669	42.9/28.6/28.6	10.6
cM						
M0	Reference				55.0/35.0/35.0	13.3
M1	3.117	0.737	13.192	0.122	50.0/0.0/0.0	8.8
Tumor location						
Lt	Reference				60.0/30.0/30.0	13.7
Mt	1.079	0.357	3.260	0.893	44.4/33.3/33.3	11.0
Ut	1.094	0.242	4.935	0.907	66.7/33.3/33.3	14.7
Albumin						
<4.0 g/dL	Reference				50.0/33.3/33.3	11.8
≥4.0 g/dL	0.849	0.273	2.640	0.777	56.3/31.3/31.3	13.2
WBC counts						
≤8000/mm^3^	Reference				66.7/40.0/40.0	14.7
>8000/mm^3^	2.473	0.850	7.199	0.097	28.6/14.3/14.3	8.7
NSE level						
≤15 ng/mL	Reference				66.7/33.3/33.3	14.3
>15 ng/mL	1.114	0.365	3.401	0.850	50.0/37.5/37.5	12.3

Abbreviation: NR, not reached.

**Table 3:** Univariate analysis for OS with Cox regression models and summary statistics

Variable	Univariate analysis (N = 22)					
	HR	95% CI		*P*‐value	1/3/5 y ‐ PFS [%]	Median [mo]
CRT regimen						
CE‐RT	Reference				81.3/66.2/46.3	37.5
CF‐RT	1.941	0.607	6.205	0.264	100.0/40.0/40.0	29.3
Age						
<65	Reference				91.7/44.4/33.3	29.3
≥65	0.584	0.173	1.975	0.387	77.8/77.8/62.2	71.2
Gender						
Male	Reference				86.7/50.0/33.3	30.7
Female	0.225	0.036	1.398	0.109	83.3/83.3/83.3	NR
PS						
0	Reference				90.9/56.6/56.6	71.2
1	1.075	0.322	3.595	0.906	80.0/60.0/37.5	34.8
cT						
T1‐2	Reference				100.0/100.0/100.0	NR
T3‐4	9.131	0.480	173.752	0.141	81.3/48.6/33.3	29.3
cN						
N0‐1	Reference				92.9/60.2/51.6	71.2
N2‐3	1.215	0.367	4.024	0.749	71.4/57.1/38.1	37.5
cM						
M0	Reference				89.5/65.2/50.3	71.2
M1	15.144	2.095	109.487	0.007	50.0/0.0/0.0	10.4
Tumor location						
Lt	Reference				100.0/66.7/53.3	71.2
Mt	1.321	0.364	4.793	0.672	75.0/45.0/45.0	24.8
Ut	1.903	0.384	9.434	0.431	66.7/66.7/33.3	32.1
Albumin						
<4.0 g/dL	Reference				60.0/30.0/30.0	29.3
≥4.0 g/dL	0.652	0.179	2.376	0.517	93.8/66.3/49.1	37.5
WBC counts						
≤8000/mm^3^	Reference				92.9/62.9/55.0	71.2
>8000/mm^3^	2.200	0.616	7.858	0.225	71.4/53.6/26.8	37.5
NSE level						
≤15 ng/mL	Reference				91.7/72.2/61.9	71.2
>15 ng/mL	1.626	0.492	5.377	0.426	87.5/46.9/23.4	24.8

Abbreviation: NR, not reached.

### 3.4 Disease progression pattern and treatment after initial relapse

A total of 8 of 22 (36.4%) patients achieved progression‐free survival, while the remaining 14 patients experienced disease progression after dCRT. Locoregional progression (LP) was observed in 4 patients, distant progression (DP) in 7, and LP + DP in 3. In 6 out of 10 DP patients, progression sites were liver or distant lymph node metastases (others in bone, skin, adrenal gland, or brain).

A total of 3 out of 14 patients received best supportive care after disease progression. Two of 4 patients with only LP received salvage esophagectomy and achieved R0 resection, but unfortunately both of them died due to tumor relapse and late radiotherapy‐related toxicity (pleural and pericardial effusion). Only 1 patient received CRT for oligometastasis on an abdominal lymph node, but died due to multiple liver metastases. The remaining 8 patients (2 with LP, 4 with DP and 2 with LP + DP) received subsequent chemotherapy, and most of them were treated with irinotecan or amrubicine.

### 3.5 Toxicity of dCRT

Toxicity profile by treatment regimen is shown in Table 4. In summary, hematological toxicity and febrile neutropenia were more common in patients treated with CE‐RT.

**Table 4:** Adverse events by CRT regimen

Adverse events (CTCAE v4.0)	CE‐RT (N = 17)		CF‐RT (N = 5)	
Acute phase; hemotological	All grade (N)	≥ Grade 3 (N)	All grade (N)	≥ Grade 3 (N)
White blood cell decreased	100% (17)	100% (17)	80% (4)	40% (2)
Neutrophil count decreased	100% (17)	94.1% (16)	80% (4)	40% (2)
Anemia	82.4% (14)	23.5% (4)	40% (2)	20% (1)
Platelet count decreased	94.1% (16)	41.2% (7)	20% (1)	20% (1)
Febrile neutropenia	—	47.1% (8)	—	20% (1)

Adverse events (CTCAE v4.0)	CE‐RT (N = 17)		CF‐RT (N = 5)	
Acute phase; non‐hemotological	All grade (N)	≥Grade 2 (N)	All grade (N)	≥Grade 2 (N)
Anorexia	88.2% (15)	35.3% (6)	100% (5)	60% (3)
Nausea	82.4% (14)	17.6% (3)	80% (4)	20% (1)
Vomiting	17.6% (3)	0% (0)	0% (0)	0% (0)
Fatigue	88.2% (15)	29.4% (5)	100% (5)	20% (1)
Mucositis oral	17.6% (3)	11.8% (2)	20% (1)	0% (0)
Diarrhea	0% (0)	0% (0)	40% (2)	0% (0)
Peripheral sensory neuropathy	0% (0)	0% (0)	20% (1)	0% (0)
Creatinine increased	47.1% (8)	29.4% (5)	60% (3)	20% (1)
Esophagitis	100% (17)	52.9% (9)	100% (5)	60% (3)
Dermatitis radiation	64.7% (11)	0% (0)	40% (2)	20% (1)

Adverse events (CTCAE v4.0)	CE‐RT (N = 17)		CF‐RT (N = 5)	
Late phase	All grade (N)	≥Grade 3 (N)	All grade (N)	≥Grade 3 (N)
Esophageal stenosis	11.8% (2)	0% (0)	0% (0)	0% (0)
Pneumonitis	76.5% (13)	0% (0)	60% (3)	0% (0)
Pleural effusion	41.2% (7)	5.9% (1)	60% (3)	40% (2)
Pericardial effusion	41.2% (7)	0% (0)	40% (2)	40% (2)

In terms of radiotherapy‐related toxicity, no unexpected adverse event was seen in acute phase, but 2 patients (9.1%, both in CF‐RT) were considered to be dead due to deterioration of the general condition related to non‐malignant pleural and pericardial effusion, in the late phase.

## 4 DISCUSSION

In NCCN and ENETS treatment guidelines,^1^, ^2^ surgery followed by adjuvant chemotherapy or dCRT are recommended for locally advanced NEC in reference to the treatment of small cell lung cancer. Casas et al,^7^ reported that MST was 20 months in patients who received systemic chemotherapy in addition to local treatment while was as short as 5 months in localized EsoNEC patients who were treated with local therapy alone. Deng et al^8^ reported that MST and 1/3/5‐year OS of localized EsoNEC patients who received surgery‐based treatment were 21.5 months and 75.0/33.4/28.4%, respectively. It is generally recognized that systemic chemotherapy is essential for multidisciplinary treatment as well as local therapy for localized NEC.

As for local therapy, Wong et al^9^ showed the data of National Cancer Database in the United States. They reported that more patients with locoregional EsoNEC received dCRT (63.0%) than surgery‐based multidisciplinary approach (7.4%), and multivariate analyses indicated that patients treated with a surgery‐based multidisciplinary approach had better prognosis than with dCRT (MST/3y‐OS: 44.9 months/50.5% vs 16.1 months/30.9%, HR: 0.60, 95% CI: 0.37－0.97, *P* = .04). Moreover, Xu et al^10^ reported the MST of limited‐stage EsoNEC as 28.0 months, and multivariate analyses also indicated that patients treated with a surgery‐based multidisciplinary approach had better prognosis than with dCRT (HR: 0.661, 95% CI: 0.451‐0.970). However, there seemed to be selection bias because the analyses in these reports did not include patient's condition which is important to decide the treatment approach. In our study, locally advanced EsoNEC patients treated with dCRT achieved favorable clinical outcome (CR rate 77.3%, MST: 37.5 months, and 5 year‐OS: 45.4%), showing consistent results with previous reports. Therefore, dCRT is an important treatment option for locally advanced EsoNEC.

From the view of concurrent treatment regimen, platinum plus etoposide has been recommended in reference to the treatment of small cell lung cancer.^1^, ^2^ However there has been also no data supporting this recommendation for EsoNEC. In our study, there seemed to be some differences in tumor response and survival outcome between CE‐RT and CF‐RT. Patients with long‐term progression‐free survival were observed only after CE‐RT. In “typical” locally advanced esophageal cancer, 5‐year PFS of patients treated with CF‐RT was reported as 17.8%‐56.6%,^6^, ^11^, ^12^ therefore the tumor response of CF might be different between “typical” esophageal cancer and EsoNEC. While platinum plus etoposide is the chemotherapy regimen most frequently used for advanced NEC worldwide, it is suggested that CE is a preferable chemotherapy regimen also in combination with radiotherapy for locally advanced EsoNEC.

However, it is well‐known that etoposide containing regimens are often associated with severe myelosuppression which was also observed in our study. Based on the risk of this chemotherapy regimen, its indication should be carefully judged considering patient's condition. For radiotherapy, 2 patients (9.1%, both received CF‐RT) experienced fatal late toxicity. Toxicity might be related to overdose irradiation of vital organs since one patient received radiation therapy planned using two‐dimensional CT, and another patient received 60 Gy irradiation over the entire esophagus and 40 Gy elective irradiation from bilateral cervical lymph node to the celiac node. In “typical” esophageal cancer, similar efficacy and less late toxicity compared to previous report 11 was obtained by dose reduction of irradiation with dose modification of concurrent chemotherapy.^12^ Even in EsoNEC, there must be room for improvement regarding the schedule, dose, and field of radiation. These are important future tasks to resolve.

From the results of prognostic analyses, patients with T3‐4 stage primary tumors and high WBC counts tended to have worse tumor control, while patients with SCLN metastases showed poor survival. All patients with T3‐4 disease were categorized to Stage III or more in our study. Previous reports for localized EsoNEC also indicated that the prognosis of Stage III patients was worse than that of Stage I‐II patients.^8^, ^10^ In addition, high T‐stage and high WBC counts were also associated with poor survival in our previously published data for “typical” esophageal cancer.^6^ High WBC counts were considered to be caused by systemic inflammation associated with high tumor burden as well as tumor invasion. Since SCLN metastasis is regarded as distant metastasis in the TNM classification for esophageal cancer, patients with SCLN metastasis were excluded from previous reports for localized EsoNEC.^7^, ^8^, ^9^, ^10^ In “typical” esophageal cancer, we previously found that some patients with SCLN metastasis could be cured by dCRT unlike those with distant disease. The current study showed no locally advanced EsoNEC patients with SCLN metastases achieved long‐term survival by dCRT, however only 2 patients with SCLN metastasis were included in this study. One patient experienced LR + DR after CE‐RT and died 7.2 months after initiation of dCRT due to primary disease, the other died after CF‐RT due to late toxicity. Further investigation is needed to judge whether EsoNEC patients with SCLN metastasis should be treated as localized or systemic disease.

The major limitation of this study is its retrospective nature and the limited sample size. Further investigation to establish the optimal treatment strategy for EsoNEC is required in a prospective cohort, although it is difficult to conduct randomized controlled trials because of the limited number of patients with EsoNEC.

In conclusion, definitive chemoradiotherapy can be an important treatment option for locally advanced esophageal neuroendocrine carcinoma.

## CONFLICT OF INTEREST

There was no Grant support and the authors have no conflict of interest for this study.

## AUTHOR'S CONTRIBUTIONS

Authors belonging to department‐1) contributed to this paper by treating the subjects with chemotherapy. Author belonging to department‐2) contributed to this paper by performing the statistical analysis. Authors belonging to department‐3) contributed to this paper by treating the subjects with radiotherapy. Authors belonging to department‐5) contributed to this paper by treating the subjects with surgery.
